# Author Correction: The choroid plexus is an important circadian clock component

**DOI:** 10.1038/s41467-019-13101-9

**Published:** 2019-11-20

**Authors:** Jihwan Myung, Christoph Schmal, Sungho Hong, Yoshiaki Tsukizawa, Pia Rose, Yong Zhang, Michael J. Holtzman, Erik De Schutter, Hanspeter Herzel, Grigory Bordyugov, Toru Takumi

**Affiliations:** 1 RIKEN Brain Science Institute (BSI), Wako, 351-0198 Japan; 2 Computational Neuroscience Unit, Okinawa Institute of Science and Technology, Okinawa, 904-0495 Japan; 3 Graduate Institute of Humanities in Medicine, Taipei Medical University, Taipei, 11031 Taiwan; 4 TMU-Research Center of Brain and Consciousness, Taipei Medical University, Taipei, 11031 Taiwan; 5 Laboratory of Braintime, Shuang Ho Hospital, New Taipei City, 23561 Taiwan; 6 Institute for Theoretical Biology, Charité-Universitätsmedizin and Humboldt Universität, Berlin, D-10115 Germany; 7 Department of Anatomy, Hiroshima University School of Medicine, Hiroshima, 734-8551 Japan; 8 Pulmonary and Clinical Care Medicine, Washington University School of Medicine, St. Louis, MO 63110 USA

**Keywords:** Circadian mechanisms, Oscillators

Correction to: *Nature Communications* 10.1038/s41467-018-03507-2, published online 14 March 2018.

The original version of this Article omitted the following from the Acknowledgements:

The Takeda Science Foundation.

This has now been corrected in both the PDF and HTML versions of the Article.

