# Supplementary material for: The choroid plexus is an important circadian clock component
Source: Nat Commun. 2018 Mar 14;9:1062. doi: 10.1038/s41467-018-03507-2 (PMC5852131; doi:10.1038/s41467-018-03507-2)
Supplement: Supplementary file 1 — Supplementary Information [file 41467_2018_3507_MOESM1_ESM.pdf]

# Supplementary Information:

## The choroid plexus is an important circadian clock component

Jihwan Myung, Christoph Schmal, Sungho Hong, Yoshiaki Tsukizawa, Pia Rose, Yong Zhang, Michael J. Holtzman, Erik De Schutter, Hanspeter Herzel, Grigory Bordyugov, Toru Takumi

## Supplementary Methods

### *Grid-Based Time Series Analysis of Experimental Data*

A spatio-temporal analysis of choroid plexus clock gene reporter construct expression, underlying **Supplementary Fig. 6-10**, has been done according to previously published routines[1,2]. Salt-and-pepper imaging noise has been removed from the raw image data using the (thresholded) median filter *Remove Outliers* from the ImageJ software. Subsequently, average intensities have been calculated from square regions of interests (ROIs) arranged on a grid that is aligned to the image[1]. These coarse-grained time series from each grid cell were detrended by means of a multiresolution analysis (MRA)[3] and components (details) that correspond to the circadian time scale were chosen for a further analysis of phases, amplitudes, and periods. See also the paragraph Rhythmicity score of the Materials and Methods section in the main text for a description of the MRA analysis.

Next, instantaneous phases $\theta_i(t)$ and amplitudes $A_i(t)$ of the time series $s_i(t)$ in each grid cell $i$ were calculated by means of an analytic signal approach[4]. The analytic signal $z(t) = s(t) + iH(s(t))$ for the discrete time series $s_i(t)$, with $H(s(t))$ being the Hilbert transform, was calculated by means of the *SCIentific PYthon* function *hilbert*. Based on the numerically evaluated $z(t)$, the instantaneous phase and amplitude at time $t$ can be obtained by $\theta(t) := \text{atan2}(\Im(z(t)), \Re(z(t)))$ and $A(t) := \sqrt{\Re(z(t))^2 + \Im(z(t))^2}$, respectively, with $\Re(z(t))$ and $(\Im(z(t))$ being the real and imaginary part of $z(t)$, respectively[4,5]. In addition to the instantaneous amplitude at each time point $t$, we also evaluated the average of the instantaneous amplitude $A_i(t)$ as well as the root-mean-square (RMS) amplitude of the time series $s_i(t)$.

Periods of each time series $s_i(t)$ were determined in a two-step process. First, initial guesses for the periods of $s_i(t)$ were made by means of an FFT analysis. Corresponding amplitudes were estimated by the RMS amplitude of the time series $s_i(t)$. In a second step, the estimated periods were fine-tuned by fitting a cosine function $A \cos(2\pi/\tau + \varphi)$ to the time series data, using the previously estimated periods and amplitudes as initial conditions for the optimization problem. Minimization of least squares employed the *curve_fit* algorithm of *SCIentific PYthon*.

Note that all three observables of interest, i.e., the period, the average instantaneous amplitude and the RMS amplitude were evaluated in the time interval $1 \text{ d} \leq t \leq 5 \text{ d}$ of each experiment or experimental condition, in order to neglect edge effects at the beginning and the end of the analytic signal. Throughout **Supplementary Fig. 6-10**, we considered only those time series in which the corresponding grid cells were in an image region where choroid plexus tissue could be identified throughout the whole course of the experiment, as determined on the basis of the bioluminescence intensity.

Finally, the Kuramoto order parameter or synchronization index $R(t)$ at time $t$ as depicted in **Supplementary Fig. 9B**, was determined from the absolute value of the complex order parameter $R(t)\, e^{i\psi(t)} := \frac{1}{N}\sum_{j=1}^{N} e^{i\theta_j}$, where $\psi(t)$ is the average phase of the ensemble at time $t$ and $\theta_i(t)$ is the instantaneous phase of time series $s_i(t)$ as described above[2].

### *RT-qPCR for quantifying homeostatic expression*

The CP sampling, culture, and total RNA purification methods are as described in the main text. CP tissues were cultured in separate dishes under four different culture conditions: medium containing 30μM 18-β-glycyrrhetinic acid (bGA), 1μM halothane, 1μM tetrodotoxin (TTX), or no inhibitor (control). After 5 days in culture, two cultured explants from two different animals were pooled for qPCR quantification for the same culture condition. For all cultured explants, RNA sampling was done at the same Zeitgeber time (ZT) 20±1. Both GAPDH and β-actin (ACTB) were used as double internal control[6]. This experiment was independently performed two times. Results are shown in **Supplementary Fig. 14**. Primers used are, in addition to those specified in the main text, as follows. Actb: GACCTCTATGCCAACACA (forward), TCCACATCTGCTGGAAGGT (reverse); mPer2: GGCTTCACCATGCCTGTTGT (forward), GGAGTTATTTCGGAGGCAAGTGT (reverse); Avp: GCTCTCCGCTTGTTTCCTGA (forward), TGGGCAGTTCTGGAAGTAGCA (reverse); V1aR1: CAGTGAAGATGACCTTTGTG (forward), ATATGCGGCTCAAGTGGAGACAG (reverse); V1aR2: TCGTCCAGATGTGGTCAGTC (forward), TGCTATCCGAGTCATCCTTG (reverse); Vip: GCCTCTCTTTGGACCACCTT (forward), CTCCTTCAAACGGCATCCT (reverse); Vpac2: TCTCTGTGCTGGTCAAGGACAG (forward), TGGAAGAATACCAGGCTGAGCT (reverse); Cx36: AAGGCATCTCCCGCTTCTACA (forward), GCCAACCAGGAACCCAATTT (reverse); Cx43: GATCGCGTGAAGGGAAGAAG (forward), CAGCCATTGAAGTAAGCATATTTTG (reverse).

## Supplementary Discussion

The twist can be understood as a case where frequency depends on coupling because a higher-frequency oscillator will have a larger amplitude and a stronger influence on its coupled oscillators. Oscillator networks where coupling strength depends on frequencies attracted a lot of attention recently since they exhibit so-called 'explosive synchronization,' where synchronization emerges through the first-order phase transition as the coupling strength increases[7,8]. Although networks in these studies have much more extensive connectivity, it is possible that a similar principle still applies to synchronization in the CP. In that case, synchronization of the CP network can be governed by the process of nucleation and growth, just as well-known examples of the first-order phase transition such as boiling water. Indeed, we observed distinctive spatial patterning in experimental data, and simulations suggest that it can be a signature of this mechanism. Particularly, in a simulation where coupling strength is gradually increased from zero, global synchronization initiates with groups of locally synchronized oscillators that generate spatial patterning, while this feature is lacking in the control case without twist (**Supplementary Fig. 13**). The twist in the CP is a novel phenomenon, unseen in previous studies on gap junction-mediated synchronous oscillations, such as the pancreatic islet model[9] where synchronization arises in a similar way to percolation and therefore is a second-order transition. In future studies, it would be interesting to explore how the twist mechanism contributes to control of synchronization in non-neuronal networks, where local coupling dominates.

## Supplementary Figures

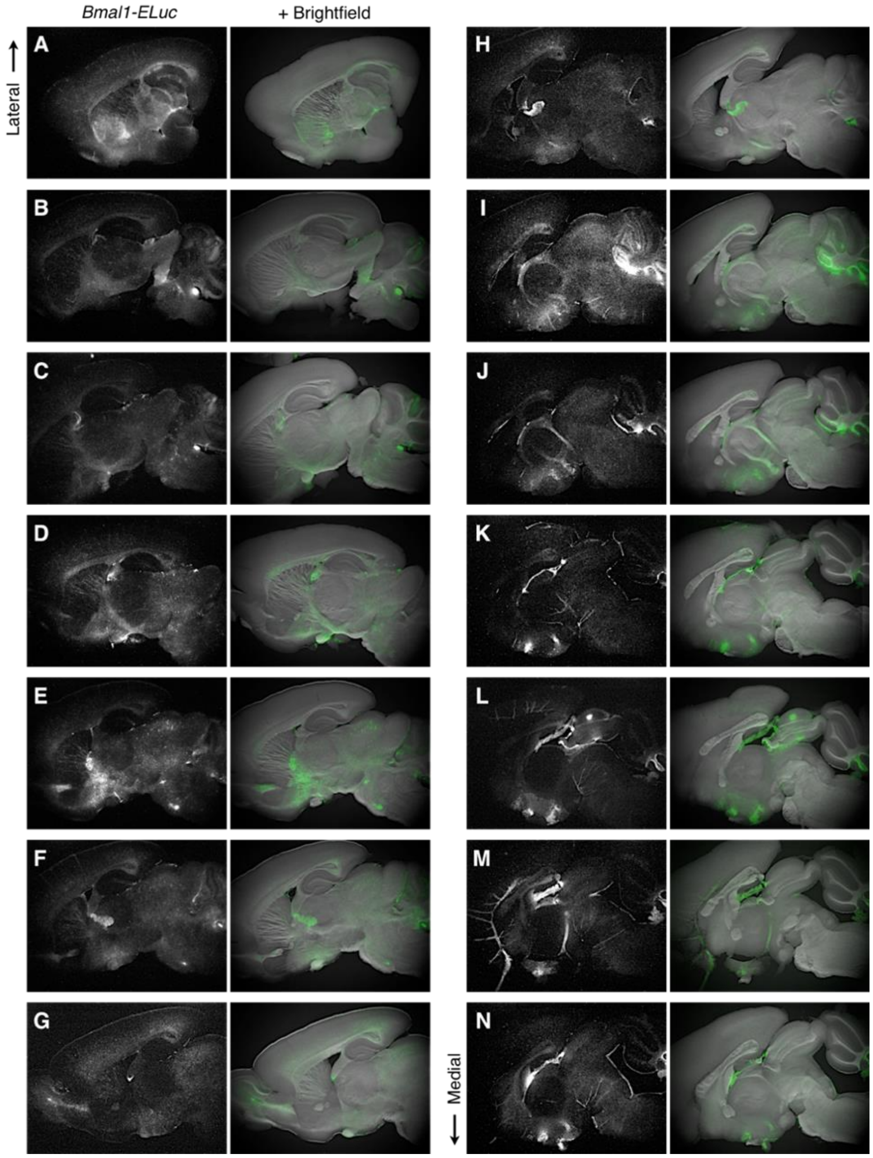


**Supplementary Figure 1.** ***(Related to Figure 1A)*** **Brain-wide expression of Bmal1-ELuc.** Serial sagittal sections (150 µm thick) from the lateral to the medial side reveal potential loci for autonomous clocks: (**A**) Basomedial amygdala (BMA), (**B**) nucleus of lateral leminscus (NLL) and dorsal cochlear nucleus (DCO), (**C**) medial amygdala nucleus (MEA), (**D**) magnocellular nucleus (MA), (**E**) superior olivary complex (SOC), reticular nucleus of the thalamus (RT), and lateral pre-optic area (LPO), (**F**) SOC and choroid plexus of the lateral ventricle (LV CP), (**G**) olfactory tract, (**H**) CP-LV and choroid plexus of the fourth ventricle (LV CP), (**I**) cerebellum and retrochiasmatic area (RCh), (**J**) dorsomedial hypothalamus (DMH), (**K**) arculate hypothalamic nucleus (ARH) and suprachiasmatic nucleus (SCN), (**L**) anteriorventral periventricular nucleus (AVPV), SCN, median eminence (ME), and ARH, (**M**) LV CP, 4V CP, SCN, unidentified tracts, and ependymal cell layer, (**N**) LV CP, SCN, ME, and DMH.

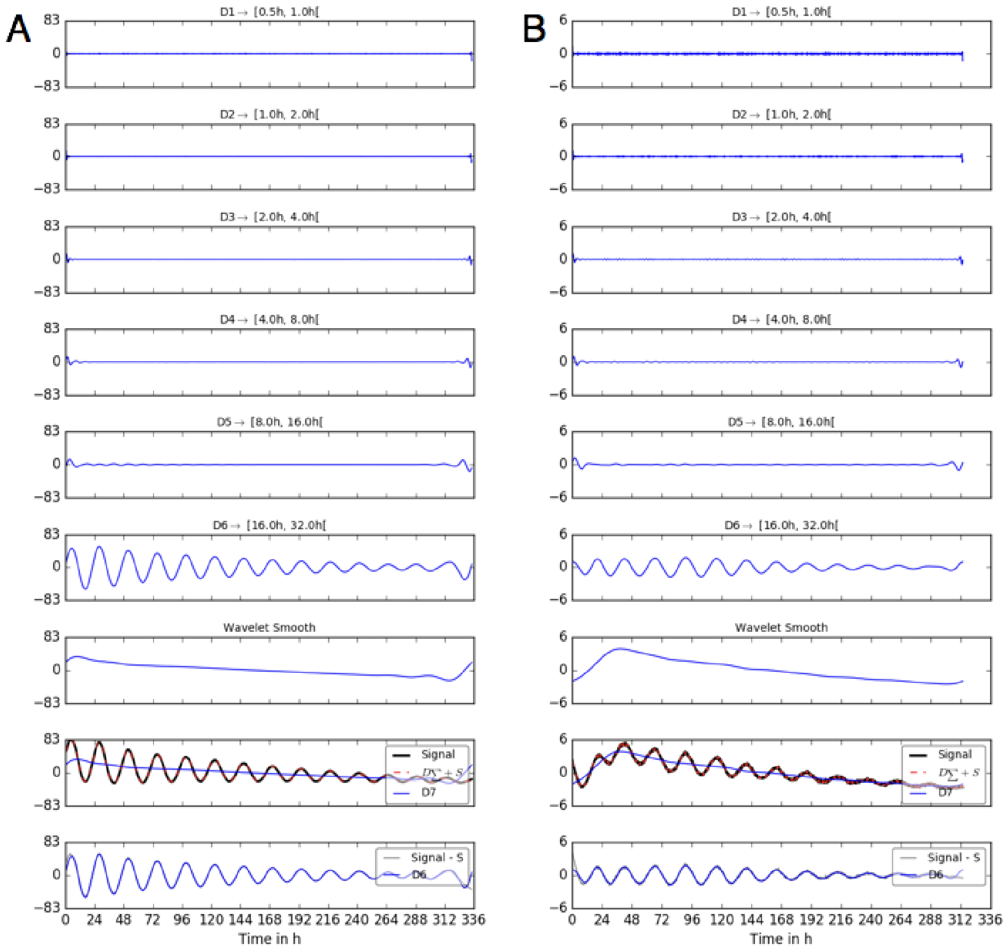


**Supplementary Figure 2.** ***(Related to Figure 1D)*** **Representative plots of spectral components *D* decomposed for the rhythmicity estimation.** Raw PER2::LUC oscillations (black trace in the panel second from bottom) are detrended by wavelet smoothing (third from the bottom) to extract the detrended oscillation (bottom). The discrete wavelet transform decomposes the oscillation into spectral intervals of 6 levels. $D_6$ (sixth from top) contains the circadian component and $D_1$ (top) is the noise component (see Materials and Methods). Representative samples are (**A**) 4V CP and (**B**) SCN, monitored on the PMT (adult mice, P38 and P21).

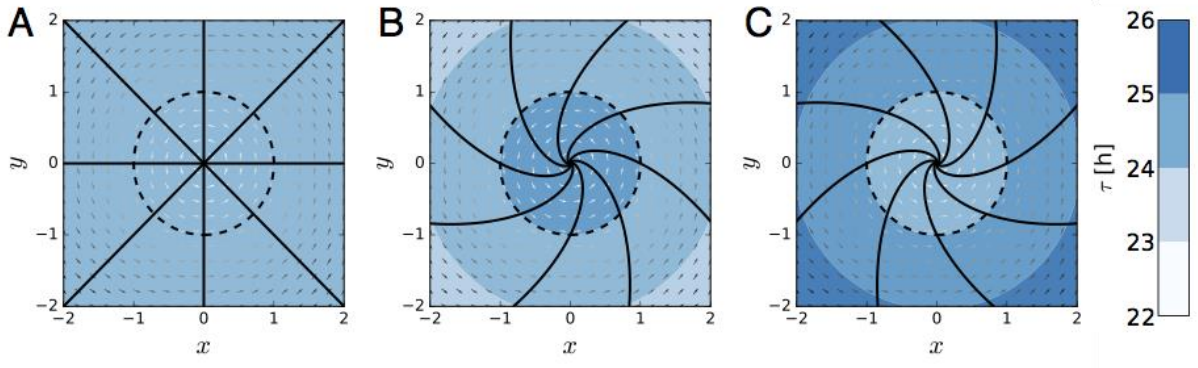


**Supplementary Figure 3.** ***(Related to Figure 2D)*** **Isochrons of the modified Poincaré oscillator.** (**A**) $\varepsilon$ = 0.0, (**B**) $\varepsilon$ = -0.01, (**C**) $\varepsilon$ = 0.01 $h^{-1}$. For all simulations, $A$ = 1, $\lambda$ = 0.02 $h^{-1}$, and $\tau$ = 24 h (see "Isochrons of the modified Poincaré model" in the Materials and Methods section of the Main Text). The dashed line indicates the steady-state limit cycle. The bold black line indicates isochrons, and arrows represent the vector space. The color-coded contour plot shows the distribution of period at a certain radius $r$.

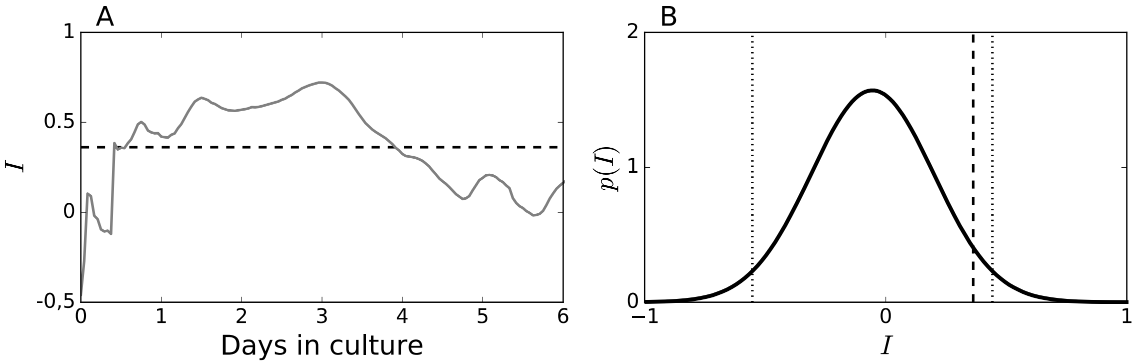


**Supplementary Figure 4.** ***(Related to Figure 2E)*** **Bmal1-ELuc oscillations show statistically significant spatial patterning of phases.** (**A**) Dynamical evolution of Moran's $I$ for phase dynamics depicted in Figure 2E. Time-dependent phase values have been determined by dimensional embedding of the detrended bioluminescence time series. The dashed line indicates a statistically significant level ($p < 0.05$, one-sided). (**B**) Sampling distribution of $I$ values under the null hypothesis of no spatial autocorrelation for a one-dimensional chain with $N$=12 elements. The histogram (gray bars) has been obtained by determining $I$ for $10^6$ randomly generated phase patterns. A Gaussian fit (bold black line) describes the sampling data well. Dashed and dotted black lines correspond to the critical values of $I$ at a significance level of 0.05 in a one- or two-sided test, respectively. The null hypothesis of no spatial autocorrelation can be withdrawn for $I$ values that exceed these lower or upper critical values.

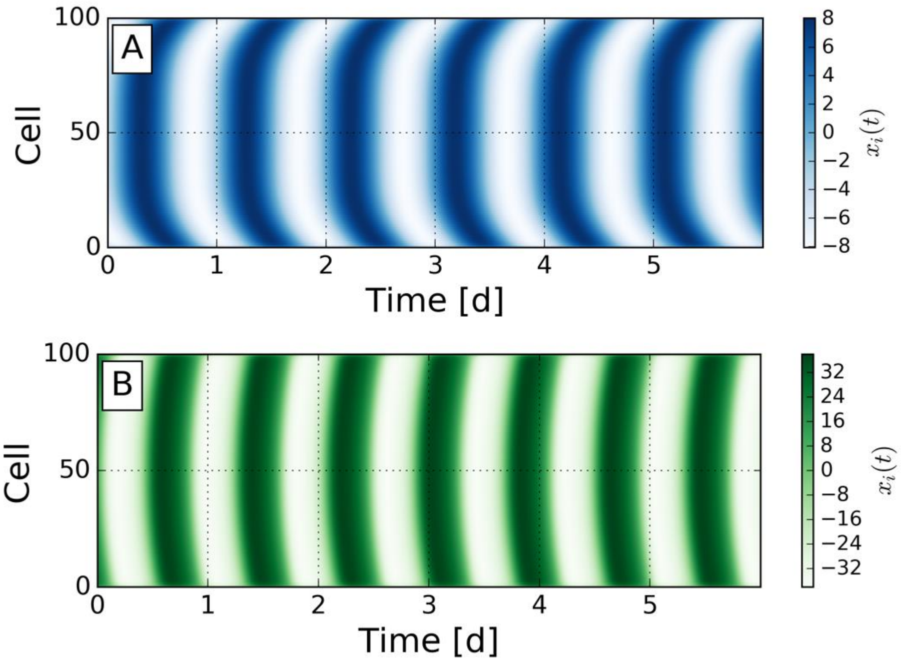


**Supplementary Figure 5.** ***(Related to Figure 2E)*** **Interdependency of amplitude, period and phase in a large linear chain of nearest-neighbor-coupled twist oscillators.** $N$ = 100 modified Poincaré oscillators with $A$ = 1, $\lambda$ = 0.02 $h^{-1}$, $\varepsilon$ = -0.01 $h^{-1}$ and $\tau$ = 25.5 h and nearest neighbor interactions of strength $K$ = 0.1 (**A**) and $K$ = 0.5 (**B**), as given by Equations (12a, 12b) of the main text, are analyzed. A similar phase-patterning as discussed for a linear chain of $N$ = 12 oscillators can also be observed for the large chain. The chain geometry in combination with nearest-neighbor coupling leads to a lower cumulative coupling strength at the edges of the chain, which in turn, leads to an earlier phase of central oscillators. Additionally, the inverse amplitude-period correlation, characteristic for a negative twist parameter $\varepsilon < 0$ can be observed: For the overcritical (i.e., all oscillators run at the same pace) coupling strength of $K$ = 0.1 (A), an 8-fold amplitude expansion of the individual oscillators amplitude ($A$ = 1) can be observed, which is accompanied by a decreasing period to a value of $\tau \sim$ 23.0 h. A further increase of the coupling strength to a value of $K$ = 0.5 (B) leads to a further increase of the oscillators amplitude and hence to a further decrease of the oscillation period to a value of $\tau \sim$ 19.5 h.

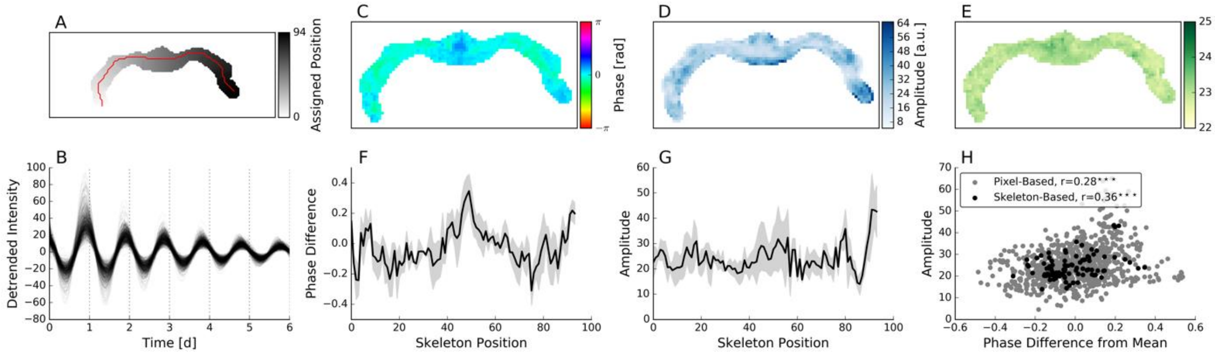


**Supplementary Figure 6.** ***(Related to Figure 3)*** **Grid analysis of two-dimensional time-lapse recordings of CP clock gene reporter expression. (A)** The gray shaded area depicts the region where CP tissue was present throughout the two-dimensional time lapse recording of CP PER2::LUC expression. The red line depicts the "skeleton" or back-bone of this structure as determined by the *Skeletonize* function of the ImageJ software. (**B**) Detrended bioluminescence intensity for each grid cell throughout the entire experiment. (**C-E**) Phase and amplitude values at time $t$ = 1.5 d as well as the oscillation period are shown for each grid element, respectively. (**F, G**) In order to test whether phases and amplitudes differ with respect to their position in the CP structure, each pixel is assigned and grouped to its closest "skeleton" position, i.e., the red line in panel (A). The black lines and gray shaded areas depict the mean values and standard deviations of the instantaneous phases (F) and amplitudes (G) at time $t$ in each of such groups of pixels. As in Figure 3E (top) of the main text, an earlier phase of oscillations can also be seen in this grid-based two-dimensional analysis. (**H**) Amplitude and mean-centered phases show a significant positive correlation, i.e., phases with a high amplitude tend to have earlier phases.

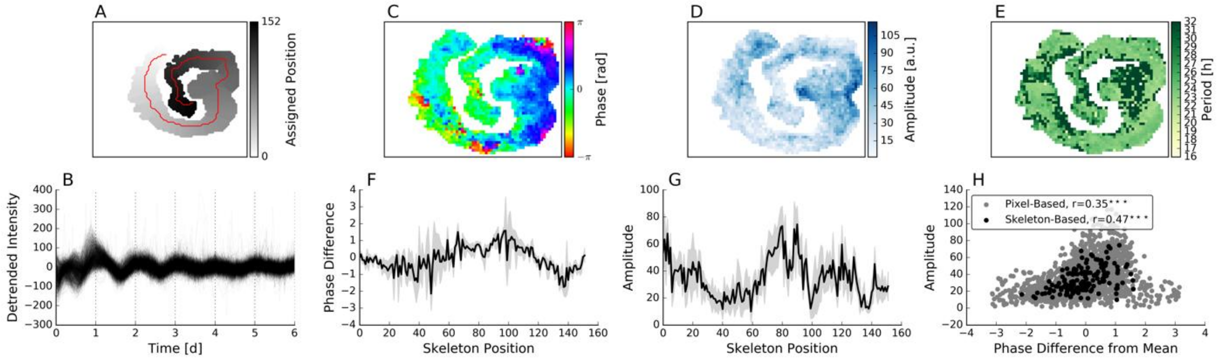


**Supplementary Figure 7.** ***(Related to Figure 3)*** Same as Supplementary Fig. 6 for an independent experimental realization. Notice that due to the camera setup, time series in this experiment are much noisier than those shown in Supplementary Fig. 6. In panels (F) and (G), the gray shades represent standard deviations.

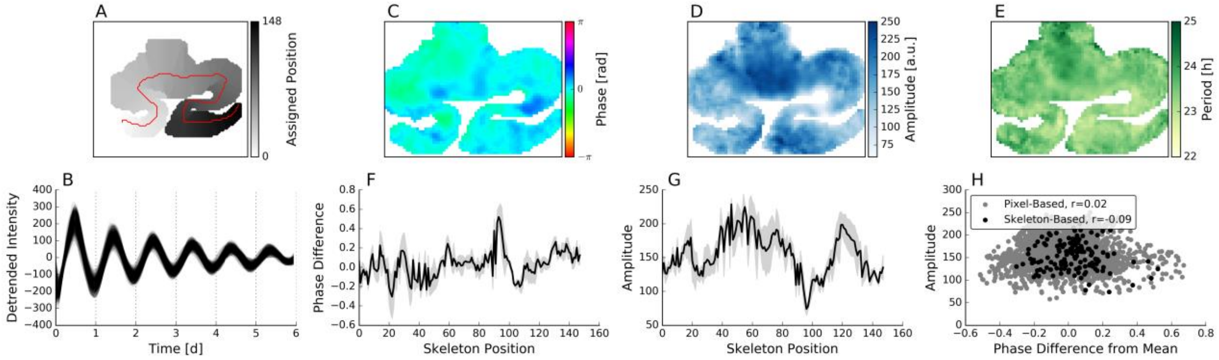


**Supplementary Figure 8.** ***(Related to Figure 3)*** Same as Supplementary Fig. 6 for an independent experimental realization. Note that in this sample no phase advancement can be seen in the center of the CP nor is there a significant correlation between the amplitude and the mean-centered phase. In panels (F) and (G), the gray shades represent standard deviations.

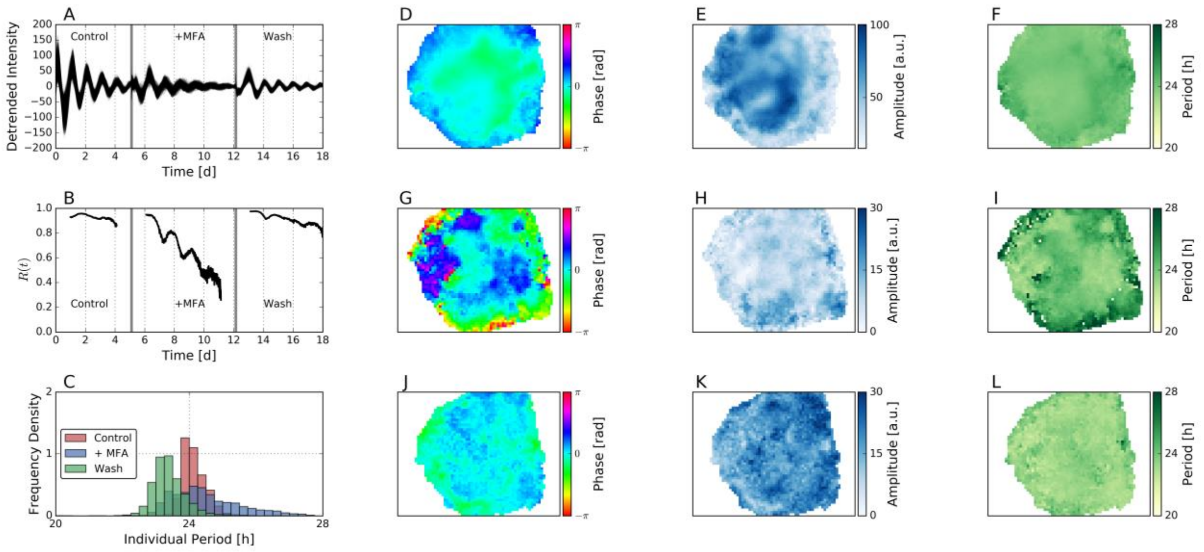


**Supplementary Figure 9.** ***(Related to Figure 3)*** **MFA reversibly desynchronizes 4V choroid plexus circadian oscillations.** (**A**) Detrended PER2::LUC bioluminescence recordings before the application of MFA (“Control”), during the application of 200 μM MFA (“MFA”), and after washing MFA from the culture medium (“Wash”). Time points of the MFA application and the washing procedure are depicted in bold gray vertical lines, respectively. (**B**) The Kuramoto order parameter $R(t)$, which is equivalent to the global phase coherence ($R = 0$ indicates incoherence and $R = 1$ a perfectly coherent network state, respectively), is plotted against time $t$ under all three conditions. In order to remove boundary effects during the phase estimation procedure, the first and last day during each experimental period were ignored. It can be observed that the global phase coherence is reversibly reduced during the application of MFA. (**C**) Period distributions under all three experimental conditions. Period distributions broaden significantly and reversibly during application of MFA. Such broadening of the period distribution can be associated with a network state of reduced inter-cellular coupling[2]. (**D-L**) Spatial phase, period, and amplitude distributions before the application of MFA (D-F), during the application of MFA (G-I), and after washing MFA from the culture medium (J-L). Instantaneous phases and amplitudes have been determined 2 days after transfer to culture medium (D, E), 4 days after the application of MFA (G, H) and 2 days after washing MFA from the culture medium (J, K).

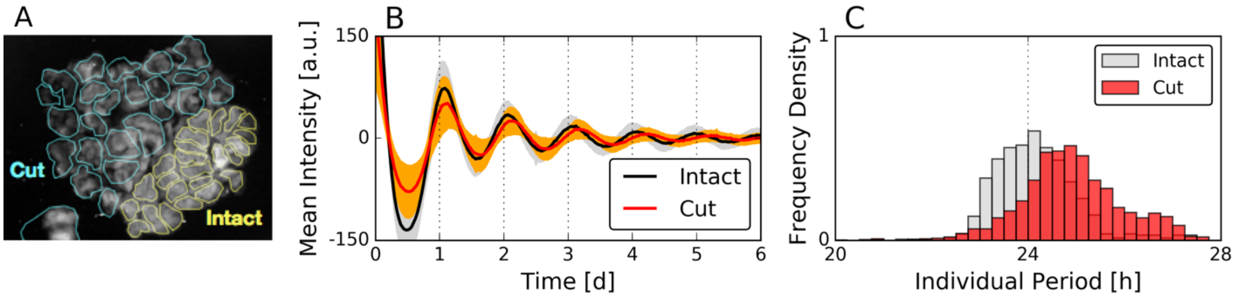


**Supplementary Figure 10.** ***(Related to Figure 3)*** **Surgical dissection of the 4V CP increases single-cell oscillation rhythms.** (**A**) Bright-field microscopy image. Regions of interest (ROIs) are associated with a surgically dissected (cyan) and an intact part (yellow) of a 4V CP. (**B**) Mean and standard deviations of the detrended time series from the ROIs associated with the intact (black curve and gray shaded area) and the dissected (red line and orange shaded area) tissue. (**C**) Period distributions of grid-cell based oscillations of the intact (gray) and dissected (red) tissue. Surgical dissection of the 4V CP leads to an increased single-cell oscillation period when compared to intact tissue. This is analogous to the case of ensemble rhythms in Figures 3E and 3F.

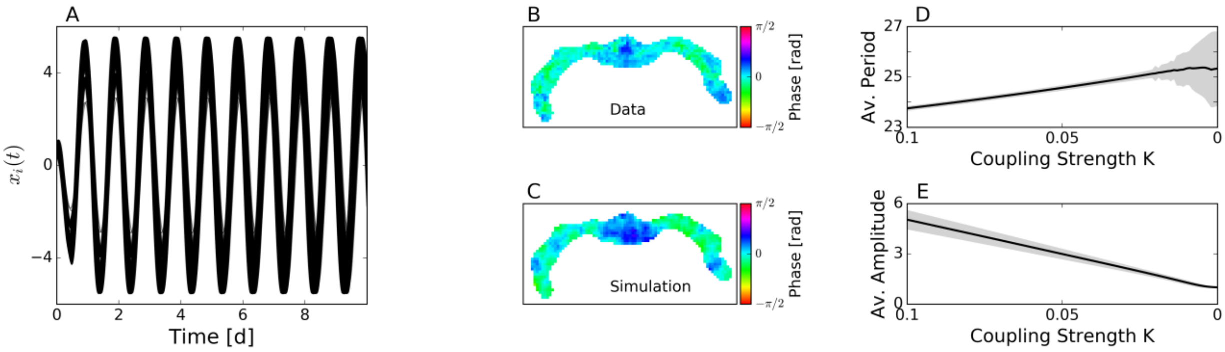


**Supplementary Figure 11.** ***(Related to Figure 2E)*** **Simulations of two-dimensional systems of nearest-neighbor coupled Poincaré oscillators can reproduce the experimentally observed phase patterns**. A two-dimensional lattice of nearest-neighbor coupled Poincaré oscillators on a "realistic" geometry is analyzed. A total of $N$ = 764 grid cells were placed with respect to the geometry as given by the experimental system in Supplementary Fig. 6. Single-cell oscillator properties were chosen as $A_i = 1$, $\lambda_i = 0.05\ h^{-1}$ and $\varepsilon_i = -0.01\ h^{-1}$. Intrinsic periods were drawn from a normal distribution with mean 25.5 h and standard deviation of 1 h. For an appropriate set of initial conditions, the system quickly reached a stably synchronized solution (see panel (**A**)) that exhibits a phase pattern similar to the pattern observed in the experimental setup, compare panels (**B**) and (**C**). Phases are centered around their mean values. Although a similar phase can be also reproduced with a two-dimensional lattice of Poincaré oscillators without twist (data not shown), the characteristic coupling, amplitude and period relationship as discussed in the main text, and Figures 3 and 4 can only be reproduced by a model exhibiting twist, compare panels (**D**) and (**E**).

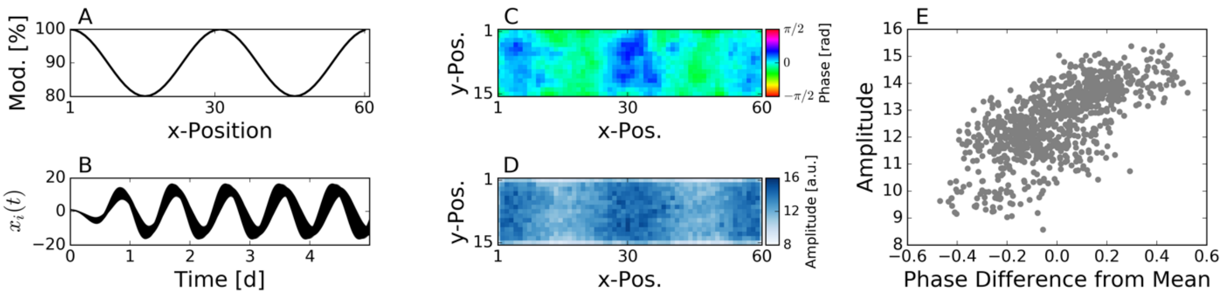


**Supplementary Figure 12.** ***(Related to Figures 2E)*** **A spatial gradient in nearest neighbor coupling strength leads to a robust phase patterning.** An ensemble of locally coupled Poincaré oscillators on a two-dimensional lattice has been simulated in case where nearest neighbor coupling strength is modulated between 80 and 100 percent of its nominal value $K$ = 0.1 with respect to the $x$-position on the lattice as indicated by panel (**A**). According to previous simulations, single-cell oscillator properties of $A_i = 1$ and $\varepsilon_i = -0.01$ h$^{-1}$ were chosen. In order to introduce single-cell variability, intrinsic periods $\tau_i$ and relaxation rates $\lambda_i$ were chosen from normal distributions with mean $<\tau>$ = 25.5 h and $<\lambda>$ = 0.02 h$^{-1}$ and standard deviations of $\sigma_\tau$ = 1 h and $\sigma_\lambda$ = 0.001 h$^{-1}$, respectively. (**B**) A stably synchronized solution is reached after short transient dynamics. (**C, D**) The spatial gradient in coupling strength leads to a stable phase pattern. Higher coupling strength in the central and lateral part leads to higher amplitude expansion upon synchronization. In combination with negative twist, this amplitude expansion leads to a shorter period of the single cell which ultimately leads to an earlier phase of the faster cells in the fully synchronized state (here shown at time point $t$ = 4 d in panel C). Hence, a positive correlation exists between the phase difference from the mean and amplitude (**E**). Note that without the assumption of twist, such robust phase patterning could not be observed in the corresponding simulations (data not shown).

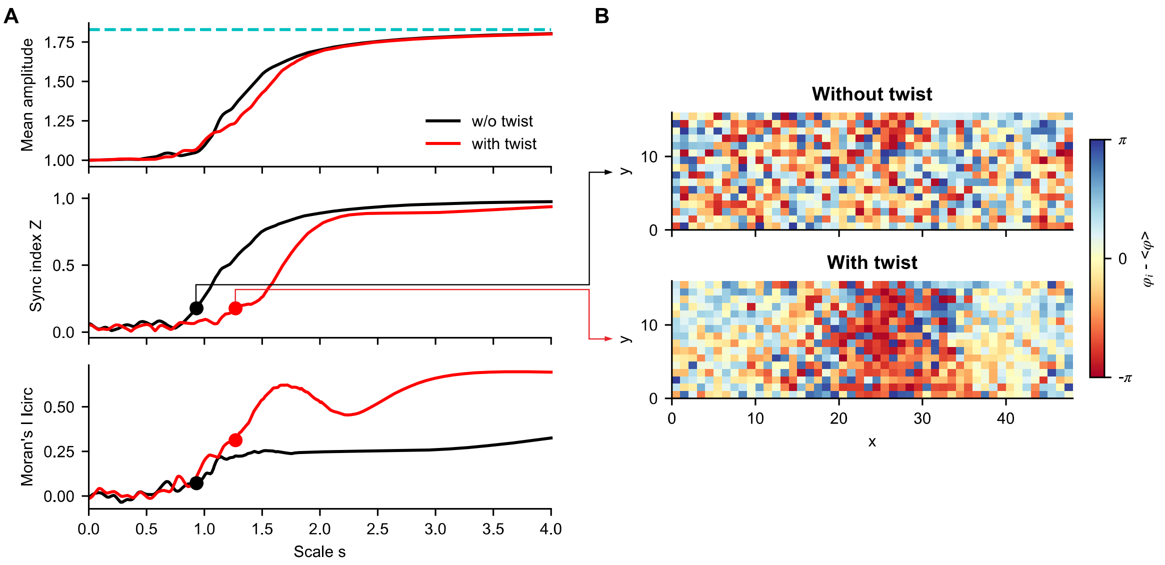


**Supplementary Figure 13.** ***(Related to Figures 2D, 2E, and 3)*** **Simulation of coupled oscillators with adiabatic increase of $\lambda$ and coupling strength.** (**A**) Mean amplitude (top), synchronization index (middle), and circular Moran's $I_{\mathrm{circ}}$ (bottom) from a simulation of coupled Poincaré oscillators. Oscillators are placed in the 48×16 grids and were coupled to each other when the mutual distance was less than four grids. $K_i = 0.0005s$ and $\lambda = 0.025s$ while scale $s$ slowly increased from 0 to 5 during 8 months of simulation time. In a model without (black) and with twist (red), $\varepsilon = 0$ and $\varepsilon = -0.02$, respectively. A blue-dotted line on top represents a prediction of the amplitude under complete synchrony. Black and red dots (middle and bottom) represent the network state for an untwisted and twisted oscillator network shown in (B), respectively. At these points, both networks have the same synchronization index $Z = 0.178$ (middle), but Moran's $I_{\mathrm{circ}}$ is higher for the twisted case (bottom). (**B**) Difference between the phases of individual oscillator ($\varphi_i$) and a mean oscillation phase ($<\varphi>$). The network states in the untwisted and twisted cases correspond to the black and red points in (A). Note that the twisted case has a blob of locally synchronized oscillators that correspond to the higher Moran's $I_{\mathrm{circ}}$ in (A), whereas synchronized oscillators are scattered around the network in the untwisted case.

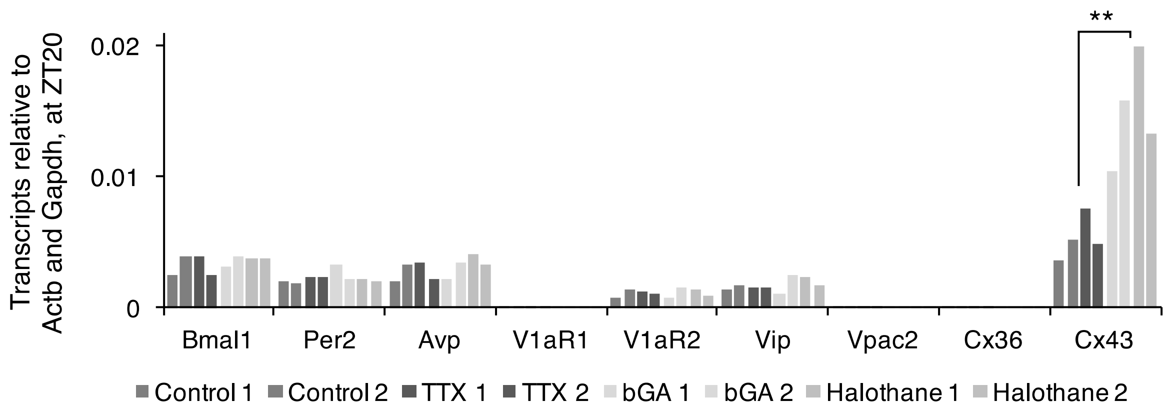


**Supplementary Figure 14.** ***(Related to Figures 2 and 3)*** **Homeostatic expression of connexin 43 (Cx43) in the cultured CP after prolonged inhibition.** Cx43 expression increased significantly after 5 days of gap junction inhibition by 30μM 18-β-glycyrrhetinic acid (bGA) or 1μM halothane in culture, while the action potential blocker 1μM tetrodotoxin (TTX) had no effect ($^{**}p<0.01$; control and TTX groups vs bGA and Halothane groups; Student's *t*-test). Two independent experiments were performed (indicated by 1 and 2). In each experiment, two CP explants from two animals were pooled for each group (control, TTX, bGA, and Halothane). Samples were taken at Zeitgeber time (ZT) 20±1 and total RNA was micro-column purified[6]. No significant differences were found in comparison groups other than Cx43.

## Supplementary References


1 Myung, J. *et al.* Period coding of Bmal1 oscillators in the suprachiasmatic nucleus. *J Neurosci* **32**, 8900-8918, doi:10.1523/JNEUROSCI.5586-11.2012 (2012).

2 Schmal, C., Herzog, E. D. & Herzel, H. Measuring relative coupling strength in circadian systems. *J Biol Rhythms* **33**, 84-98, doi:10.1177/0748730417740467 (2017).

3 St John, P. C. & Doyle, F. J., 3rd. Quantifying stochastic noise in cultured circadian reporter cells. *PLoS Comput Biol* **11**, e1004451, doi:10.1371/journal.pcbi.1004451 (2015).

4 Schmal, C., Myung, J., Herzel, H. & Bordyugov, G. Moran's I quantifies spatio-temporal pattern formation in neural imaging data. *Bioinformatics* **33**, 3072-3079, doi:10.1093/bioinformatics/btx351 (2017).

5 Pikovsky, A., Rosenblum, M. & Kurths, J. *Synchronization : a universal concept in nonlinear sciences*. (Cambridge University Press, 2001).

6 Myung, J. *et al.* GABA-mediated repulsive coupling between circadian clock neurons in the SCN encodes seasonal time. *Proc Natl Acad Sci U S A* **112**, E3920-3929, doi:10.1073/pnas.1421200112 (2015).

7 Gomez-Gardenes, J., Gomez, S., Arenas, A. & Moreno, Y. Explosive synchronization transitions in scale-free networks. *Phys Rev Lett* **106**, 128701, doi:10.1103/PhysRevLett.106.128701 (2011).

8 Zhang, X., Hu, X., Kurths, J. & Liu, Z. Explosive synchronization in a general complex network. *Phys Rev E Stat Nonlin Soft Matter Phys* **88**, 010802, doi:10.1103/PhysRevE.88.010802 (2013).

9 Benninger, R. K., Zhang, M., Head, W. S., Satin, L. S. & Piston, D. W. Gap junction coupling and calcium waves in the pancreatic islet. *Biophys J* **95**, 5048-5061, doi:10.1529/biophysj.108.140863 (2008).
